# Supplementary material for: Modeling glioblastoma heterogeneity as a dynamic network of cell states
Source: Mol Syst Biol. 2021 Sep 16;17(9):e10105. doi: 10.15252/msb.202010105 (PMC8444284; doi:10.15252/msb.202010105)
Supplement: Supplementary file 5 — Source Data for Figure 3 [file MSB-17-e10105-s001.zip › Figure3A_sourcedata/GSEA_3065/nosigdb_state6.GseaPreranked.1622718443993/RICHARDS_NOWAKOWSKI_SCIENCE_2017_MGE_IPC3_UPREG.html]

Details for gene set RICHARDS\_NOWAKOWSKI\_SCIENCE\_2017\_MGE\_IPC3\_UPREG[GSEA]

|  || Dataset | state6 |
| Phenotype | NoPhenotypeAvailable |
| Upregulated in class | na\_pos |
| GeneSet | RICHARDS\_NOWAKOWSKI\_SCIENCE\_2017\_MGE\_IPC3\_UPREG |
| Enrichment Score (ES) | 0.3803253 |
| Normalized Enrichment Score (NES) | 1.6443095 |
| Nominal p-value | 0.0024875621 |
| FDR q-value | 0.017157698 |
| FWER p-Value | 0.321 |
Table: GSEA Results Summary

  

Fig 1: Enrichment plot: RICHARDS\_NOWAKOWSKI\_SCIENCE\_2017\_MGE\_IPC3\_UPREG      
 Profile of the Running ES Score & Positions of GeneSet Members on the Rank Ordered List

  

| PROBE | GENE SYMBOL | GENE\_TITLE | RANK IN GENE LIST | RANK METRIC SCORE | RUNNING ES | CORE ENRICHMENT || 1 | SOX4 |  |  | 3 | 0.375 | 0.0318 | Yes |
| 2 | CCND2 |  |  | 32 | 0.269 | 0.0517 | Yes |
| 3 | NDUFC1 |  |  | 37 | 0.255 | 0.0731 | Yes |
| 4 | HES6 |  |  | 44 | 0.248 | 0.0937 | Yes |
| 5 | DLL3 |  |  | 49 | 0.246 | 0.1143 | Yes |
| 6 | RPL4 |  |  | 60 | 0.237 | 0.1334 | Yes |
| 7 | RPS11 |  |  | 85 | 0.221 | 0.1497 | Yes |
| 8 | PTMA |  |  | 94 | 0.216 | 0.1673 | Yes |
| 9 | SERF2 |  |  | 97 | 0.215 | 0.1855 | Yes |
| 10 | GLCCI1 |  |  | 101 | 0.213 | 0.2034 | Yes |
| 11 | H3F3A |  |  | 104 | 0.212 | 0.2214 | Yes |
| 12 | HNRNPA1 |  |  | 108 | 0.209 | 0.2390 | Yes |
| 13 | MZT2B |  |  | 113 | 0.206 | 0.2562 | Yes |
| 14 | H1FX |  |  | 145 | 0.194 | 0.2693 | Yes |
| 15 | TMSB4X |  |  | 162 | 0.187 | 0.2835 | Yes |
| 16 | RGS16 |  |  | 239 | 0.164 | 0.2888 | Yes |
| 17 | RPL23A |  |  | 246 | 0.163 | 0.3021 | Yes |
| 18 | GNG5 |  |  | 326 | 0.147 | 0.3056 | Yes |
| 19 | HNRNPC |  |  | 331 | 0.147 | 0.3177 | Yes |
| 20 | CORO1C |  |  | 345 | 0.145 | 0.3286 | Yes |
| 21 | CITED2 |  |  | 360 | 0.142 | 0.3392 | Yes |
| 22 | HNRNPH1 |  |  | 378 | 0.139 | 0.3492 | Yes |
| 23 | YBX1 |  |  | 384 | 0.139 | 0.3605 | Yes |
| 24 | FBXW7 |  |  | 498 | 0.124 | 0.3581 | Yes |
| 25 | RPL21 |  |  | 554 | 0.118 | 0.3619 | Yes |
| 26 | HMGN2 |  |  | 679 | 0.108 | 0.3568 | Yes |
| 27 | SH3BGRL |  |  | 701 | 0.105 | 0.3634 | Yes |
| 28 | SUMO2 |  |  | 710 | 0.105 | 0.3715 | Yes |
| 29 | CDK6 |  |  | 807 | 0.097 | 0.3688 | Yes |
| 30 | PTPRN2 |  |  | 854 | 0.094 | 0.3715 | Yes |
| 31 | SOX2 |  |  | 863 | 0.093 | 0.3786 | Yes |
| 32 | PFN2 |  |  | 942 | 0.089 | 0.3773 | Yes |
| 33 | TOX3 |  |  | 999 | 0.086 | 0.3782 | Yes |
| 34 | MARCKS |  |  | 1081 | 0.081 | 0.3758 | Yes |
| 35 | RFTN2 |  |  | 1196 | 0.075 | 0.3692 | Yes |
| 36 | BAZ2B |  |  | 1208 | 0.075 | 0.3744 | Yes |
| 37 | RND3 |  |  | 1213 | 0.075 | 0.3803 | Yes |
| 38 | CKB |  |  | 1359 | 0.069 | 0.3695 | No |
| 39 | ABAT |  |  | 1484 | 0.065 | 0.3608 | No |
| 40 | TCF3 |  |  | 1619 | 0.060 | 0.3505 | No |
| 41 | STMN1 |  |  | 1649 | 0.058 | 0.3522 | No |
| 42 | USP1 |  |  | 1712 | 0.056 | 0.3498 | No |
| 43 | XPO6 |  |  | 1802 | 0.054 | 0.3442 | No |
| 44 | CHD7 |  |  | 1953 | 0.049 | 0.3311 | No |
| 45 | SREBF2 |  |  | 2018 | 0.047 | 0.3278 | No |
| 46 | INSM1 |  |  | 2051 | 0.046 | 0.3281 | No |
| 47 | TUBA1B |  |  | 2087 | 0.045 | 0.3279 | No |
| 48 | FEM1C |  |  | 2356 | 0.037 | 0.3002 | No |
| 49 | BAZ1A |  |  | 2495 | 0.033 | 0.2872 | No |
| 50 | KLHL13 |  |  | 2530 | 0.033 | 0.2861 | No |
| 51 | OXR1 |  |  | 2533 | 0.032 | 0.2886 | No |
| 52 | TSG101 |  |  | 2565 | 0.032 | 0.2878 | No |
| 53 | ANP32E |  |  | 2570 | 0.032 | 0.2900 | No |
| 54 | CERS6 |  |  | 2731 | 0.028 | 0.2740 | No |
| 55 | CHD3 |  |  | 2757 | 0.028 | 0.2735 | No |
| 56 | RNASEH2B |  |  | 2772 | 0.027 | 0.2742 | No |
| 57 | UBB |  |  | 2890 | 0.024 | 0.2628 | No |
| 58 | RAB3IP |  |  | 2911 | 0.024 | 0.2626 | No |
| 59 | ICE1 |  |  | 2942 | 0.023 | 0.2611 | No |
| 60 | TMPO |  |  | 2978 | 0.022 | 0.2590 | No |
| 61 | VEZF1 |  |  | 3022 | 0.022 | 0.2559 | No |
| 62 | OLA1 |  |  | 3029 | 0.021 | 0.2571 | No |
| 63 | PSMB2 |  |  | 3030 | 0.021 | 0.2589 | No |
| 64 | DCLK2 |  |  | 3031 | 0.021 | 0.2607 | No |
| 65 | SCYL2 |  |  | 3080 | 0.020 | 0.2569 | No |
| 66 | CDC42SE2 |  |  | 3771 | 0.005 | 0.1779 | No |
| 67 | MTAP |  |  | 3834 | 0.004 | 0.1711 | No |
| 68 | CS |  |  | 3864 | 0.003 | 0.1681 | No |
| 69 | CPSF7 |  |  | 4298 | -0.005 | 0.1186 | No |
| 70 | ILDR2 |  |  | 4444 | -0.008 | 0.1026 | No |
| 71 | EIF1AX |  |  | 4719 | -0.013 | 0.0721 | No |
| 72 | UPF3B |  |  | 4763 | -0.014 | 0.0684 | No |
| 73 | SACS |  |  | 5232 | -0.023 | 0.0164 | No |
| 74 | EPHA4 |  |  | 5330 | -0.025 | 0.0074 | No |
| 75 | ACTB |  |  | 5477 | -0.028 | -0.0070 | No |
| 76 | PBX3 |  |  | 5501 | -0.028 | -0.0072 | No |
| 77 | PPCS |  |  | 5510 | -0.029 | -0.0057 | No |
| 78 | SERINC5 |  |  | 5716 | -0.033 | -0.0265 | No |
| 79 | DCTN4 |  |  | 5895 | -0.036 | -0.0439 | No |
| 80 | CEP83 |  |  | 5921 | -0.036 | -0.0437 | No |
| 81 | NUDT1 |  |  | 6071 | -0.040 | -0.0575 | No |
| 82 | CCDC88A |  |  | 6086 | -0.040 | -0.0556 | No |
| 83 | GINS2 |  |  | 6461 | -0.049 | -0.0945 | No |
| 84 | PDCL |  |  | 6558 | -0.052 | -0.1011 | No |
| 85 | CCNE2 |  |  | 6916 | -0.062 | -0.1369 | No |
| 86 | MTMR4 |  |  | 6998 | -0.065 | -0.1407 | No |
| 87 | WDR70 |  |  | 7169 | -0.070 | -0.1543 | No |
| 88 | HMGB1 |  |  | 7186 | -0.071 | -0.1501 | No |
| 89 | TEAD1 |  |  | 7358 | -0.076 | -0.1632 | No |
| 90 | HAT1 |  |  | 7538 | -0.083 | -0.1767 | No |
| 91 | SOX6 |  |  | 7616 | -0.087 | -0.1781 | No |
| 92 | DYRK1A |  |  | 7781 | -0.094 | -0.1889 | No |
| 93 | TMEM123 |  |  | 7786 | -0.095 | -0.1812 | No |
| 94 | MAP1A |  |  | 7841 | -0.098 | -0.1790 | No |
| 95 | SF3B4 |  |  | 7887 | -0.101 | -0.1756 | No |
| 96 | ST8SIA5 |  |  | 7931 | -0.103 | -0.1717 | No |
| 97 | HNRNPA2B1 |  |  | 7941 | -0.103 | -0.1639 | No |
| 98 | CASD1 |  |  | 7961 | -0.105 | -0.1571 | No |
| 99 | MAPRE2 |  |  | 8044 | -0.110 | -0.1571 | No |
| 100 | RBL1 |  |  | 8069 | -0.111 | -0.1504 | No |
| 101 | MCM2 |  |  | 8099 | -0.114 | -0.1440 | No |
| 102 | MSI2 |  |  | 8115 | -0.115 | -0.1358 | No |
| 103 | CDCA7 |  |  | 8337 | -0.137 | -0.1495 | No |
| 104 | HMGB2 |  |  | 8388 | -0.145 | -0.1429 | No |
| 105 | TPR |  |  | 8420 | -0.149 | -0.1337 | No |
| 106 | CLSPN |  |  | 8440 | -0.151 | -0.1230 | No |
| 107 | PSMC2 |  |  | 8501 | -0.161 | -0.1160 | No |
| 108 | NUCB2 |  |  | 8584 | -0.184 | -0.1097 | No |
| 109 | NOP56 |  |  | 8619 | -0.199 | -0.0966 | No |
| 110 | NES |  |  | 8677 | -0.227 | -0.0837 | No |
| 111 | CENPF |  |  | 8703 | -0.240 | -0.0660 | No |
| 112 | HELLS |  |  | 8720 | -0.260 | -0.0455 | No |
| 113 | EPRS |  |  | 8721 | -0.260 | -0.0232 | No |
| 114 | HSPA5 |  |  | 8785 | -0.369 | 0.0013 | No |
Table: GSEA details [plain text format]

  

Fig 2: RICHARDS\_NOWAKOWSKI\_SCIENCE\_2017\_MGE\_IPC3\_UPREG: Random ES distribution      
 Gene set null distribution of ES for **RICHARDS\_NOWAKOWSKI\_SCIENCE\_2017\_MGE\_IPC3\_UPREG**
